# Correlations of Salivary Biomarkers with Clinical Assessments in Patients with Cystic Fibrosis

**DOI:** 10.1371/journal.pone.0135237

**Published:** 2015-08-10

**Authors:** Shuai Nie, Huaibin Zhang, Kathryn M. Mayer, Frank G. Oppenheim, Frédéric F. Little, Jonathan Greenberg, Ahmet Z. Uluer, David R. Walt

**Affiliations:** 1 Department of Chemistry, Tufts University, Medford, Massachusetts, United States of America; 2 Goldman School of Dental Medicine, Boston University, Boston, Massachusetts, United States of America; 3 School of Medicine, Boston University, Boston, Massachusetts, United States of America; 4 Division of Respiratory Diseases, Boston Children’s Hospital and Brigham & Women’s Hospital, Harvard Medical School, Boston, Massachusetts, United States of America; Central Michigan University School of Medicine, UNITED STATES

## Abstract

**Rationale:**

Monitoring clinical disease status in cystic fibrosis frequently requires invasive collection of clinical samples. Due to its noninvasive collection process and direct anatomic relationship with the lower airway, saliva shows great potential as a biological fluid for cystic fibrosis monitoring.

**Objectives:**

To measure the levels of multiple protein markers in human saliva supernatants and investigate the possibility of utilizing them to provide a more quantitative measure of disease state for use in research and monitoring of patients with cystic fibrosis clinically.

**Methods:**

Whole saliva samples were collected and processed from cystic fibrosis patients at two distinct time points (2010 and 2013) and measured by two separate platforms. In this cross sectional study, a convenience sample of 71 participants were recruited with samples measured by multiplexed fluorescence microarray (fiber microarray) and another 117 participant samples were measured by an automated, point-of-care, analyzer (SDReader) using a microsphere-based array via fluorescence sandwich immunoassay. For comparison, saliva from 56 and 50 healthy subjects were collected, respectively. The levels of six target proteins were quantified. Various demographic and clinical data, including spirometry, medical history, and clinicians’ assessments were also collected from patients with cystic fibrosis on the day of saliva collection.

**Measurements and Main Results:**

Similar trends were observed with both platforms and compared with healthy subjects, cystic fibrosis patients had significantly elevated levels of VEGF, IP-10, IL-8, and EGF as well as lower levels of MMP-9 (*P* ≤ 0.005) using fiber microarray and significantly elevated levels of IP-10, IL-8 with lower levels of MMP-9 and IL-1*β* (*P* ≤ 0.02) using the SDReader. The levels of the six proteins correlated with each other significantly, and in some cases, biomarker levels could be used to differentiate between subgroups of patients with different clinical presentations. For example, IP-10 levels significantly correlated with FEV_1_ and disease severity (as evaluated by clinicians) with both platforms (*P* < 0.05).

**Conclusions:**

Significant variations of the levels of six proteins in saliva supernatants, and the correlations of these levels with clinical assessments, demonstrated the potential of saliva for cystic fibrosis research and monitoring.

## Introduction

Cystic fibrosis (CF) is the most common life-threatening, genetically inherited disease for individuals of Northern European descent (1 in about 2500 newborns) [1–3]. Advancements in medical care over the last several decades have led to improvement in clinical outcomes of patients with CF, resulting in a rise of the median predicted age of survival in the US from 27 years (1986) to 38 years (2010) [4]. Despite these advancements, chronic airway infection and inflammation continue to result in significant morbidity and the prognosis for people from lower socio-economic classes lags far behind [5, 6]. It is critical to develop effective, noninvasive, and economical monitoring techniques for CF.

Currently, monitoring of CF includes sampling biofluids (*e*.*g*. oropharyngeal swab, induced sputum, serum, and bronchoalveolar lavage (BAL) fluids) that frequently require invasive and uncomfortable collection procedures, as well as expensive equipment and experienced personnel [7, 8]. Because it can be collected noninvasively by personnel with minimal training, saliva has attracted much attention in recent years as a substitute for traditional diagnostic samples [9–14]. Whole saliva and its components have already been shown to correlate with clinical disease markers in asthma [15], and its direct anatomic relationship with the lower airway may provide a window into the nature of the disease-specific response of the respiratory system in CF [16–18].

CF respiratory disease was chosen to validate the saliva diagnostic technique based on well-substantiated studies on markers of inflammation in sputum and blood [19–23]. In addition, many of these publications reported significant differences in the levels of various protein markers among patients with CF and healthy subjects. Saliva is another complex fluid and includes components of blood as well as other oropharyngeal constituents, making it distinct from sputum. A small study demonstrated altered composition of saliva reflecting impact of inflammatory process and oxidative stress on saliva and its potential role as a marker of disease [24]. Salivary electrolytes are also altered depending on various factors associated with CF [25, 26]. The aim of this study was to use a noninvasive method to measure the levels of multiple protein markers in human saliva and assess the feasibility of utilizing these markers for monitoring lung disease in CF. The SDReader, an integrated and fully automated platform with saliva loaded onto a disposable microfluidic chip, was developed concurrently to provide point-of-care (POC) analysis of saliva. The ease of access to specimen combined with prompt analysis, makes saliva an ideal biomarker. Some of the results of this study have been reported in the form of an abstract at the 2013 American Thoracic Society International Conference [27].

## Materials and Methods

### Study Design and Population

This study was approved by the Institutional Review Boards at both the CF Center at Boston Children’s and Brigham & Women’s Hospital and Goldman School of Dental Medicine of Boston University. Written informed consent was obtained from all participants or their surrogates (for children under 18).

This study was designed to investigate the possibility of using human saliva (or its components) as a sample for CF research and monitoring. All patients with CF were recruited at the CF Center at Boston Children’s and Brigham & Women’s Hospital at two distinct time points (2010 for fiber microarray and 2013 for SDReader cohorts). All patients had a confirmed diagnosis of CF (levels of sweat chloride ≥ 60 mmol/L and/or a genotype with two identifiable mutations consistent with CF, accompanied by one or more clinical features consistent with the CF phenotype). Male and female patients ≥ 6 years old were recruited. In addition, healthy subjects with no history of CF were recruited at the Goldman School of Dental Medicine of Boston University as the control group.

Medical histories of patients with CF were obtained from hospital records. All patients with CF were asked to fill out a specifically-designed questionnaire (S1 File) covering topics including lifestyle factors and self-evaluation of current symptoms. Additional clinical data including spirometry results and clinician assessment of disease status were collected via chart review.

### Saliva Sample Collection and Processing

Masticatory saliva samples were collected and processed following the protocol reported previously [28]. Briefly, after food and liquid abstinence for 30 min, participants were asked to chew a piece of Parafilm (to help saliva production) and expectorate saliva every 30 s into a 50 mL tube on ice until 10 mL of whole saliva were collected. Whole saliva samples were centrifuged at 13,150*g* for 20 min at 4°C, and the supernatant was harvested, aliquoted in 1 mL microcentrifuge tubes, transferred to Tufts on dry ice, and stored at −80°C until analyzed.

### Saliva Supernatant Analysis

The saliva supernatants were measured at room temperature using either a fiber microarray [29] or SDReader [30] as previously reported to quantify the concentrations of six proteins: human vascular endothelial growth factor (VEGF), interferon gamma-induced protein 10 (IP-10), interleukin-8 (IL-8), epidermal growth factor (EGF), matrix metalloproteinase 9 (MMP-9), and interleukin-1 beta (IL-1*β*) [28, 29].

All antibodies and recombinant human protein standards were purchased from R&D systems (Minneapolis, MN) for this study. The lower limits of detection of the assay on the fiber microarray and SDReader respectively, were as follows: VEGF, 6 and 14 pg/mL; IP-10, 26 and 30 pg/mL; IL-8, 4 and 6 pg/mL; EGF, 3 and 4 pg/mL; MMP-9, 1.3 and 8.6 ng/mL; and IL-1*β*, 5 and 82 pg/mL. For saliva samples with protein levels below the assay lower limit of detection, the corresponding protein concentrations are listed as 0.

### Statistical Analysis

The protein concentrations in different groups are presented as median (25th-75th percentile), unless stated otherwise. Comparison between different groups was performed using the nonparametric Mann-Whitney U test and the correlations were assessed using the Spearman test. Results were considered statistically significant at a value of *P* < 0.05. All statistical analyses were done using Origin Pro 9.0 (OriginLab Corporation, Northampton, MA).

## Results

### Subjects

In total, 127 subjects were recruited for the fiber microarray study and 167 subjects were recruited for the SDReader study. For the microarray study, 71 patients with CF participated, with a mean age of 23 years (range, 7–66) and 39 (55%) were female (Table 1). In addition, 56 healthy individuals were recruited for the fiber microarray study as controls, with a mean age of 32 years (range, 19–77) including 34 (61%) female subjects. For the SDReader study, 117 patients with CF were recruited to participate, with a mean age of 26 years (range, 6–67) and 58 (50%) were female (Table 2). Additionally, 50 healthy individuals with a mean age of 33 years (range, 23–77) including 30 (60%) female subjects, were recruited as the control group for the SDReader study. Our study participants are representative of CF patients in the general U.S. population, except for the prevalence of specific bacterial infections that are different amongst our subjects [4].

**Table 1:** Characteristics of patients with CF tested by the fiber microarray. F508del: F508 deletion mutation; FEV_1_, forced expiratory volume, 1s; FVC, full vital capacity; FEF, forced expiratory flow; MRSA, methicillin-resistant *Staphylococcus aureus*; PA, *Pseudomonas aeruginosa*.

Characteristics	n = 71
Mean age, yr (range)	23 (7–66)
Gender, female (%)	39 (55)
Genotype	
F508del homozygous (%)	26 (37)
F508del heterozygous (%)	31 (44)
Other (%)	14 (19)
Median FEV_1_% predicted (25%–75%)^*^	88 (65–100)
Median FEV_1_/FVC (25%–75%)^*^	0.88 (0.80–0.95)
Median FEF% predicted (25%–75%)^†^	59 (38–83)
No. of patients with infections:	
Without MRSA and PA (%)	11 (15)
With MRSA alone (%)	14 (20)
With PA alone (%)	24 (34)
With both MRSA and PA (%)	22 (31)

^*^total n = 66

^†^total n = 64

**Table 2:** Characteristics of patients with CF tested by the SDReader.

Characteristics	n = 117
Mean age, yr (range)	26 (6–67)
Sex, female (%)	58 (50)
Median FEV_1_% predicted (25%–75%)^*^	84 (55–99)
Median FVC % predicted (25%–75%)^*^	92 (71–104)
Median FEV_1_/FVC (25%–75%)^*^	0.88 (0.79–0.98)
Median FEF% predicted (25%–75%)^*^	60 (29–91)
No. of patients with infections:	
Without MRSA and PA (%)	39 (33)
With MRSA alone (%)	11 (9)
With PA alone (%)	46 (39)
With both MRSA and PA (%)	21 (18)

^*^total n = 108

### Salivary Protein Levels in Patients with CF and Healthy Subjects

**Fig 1:**
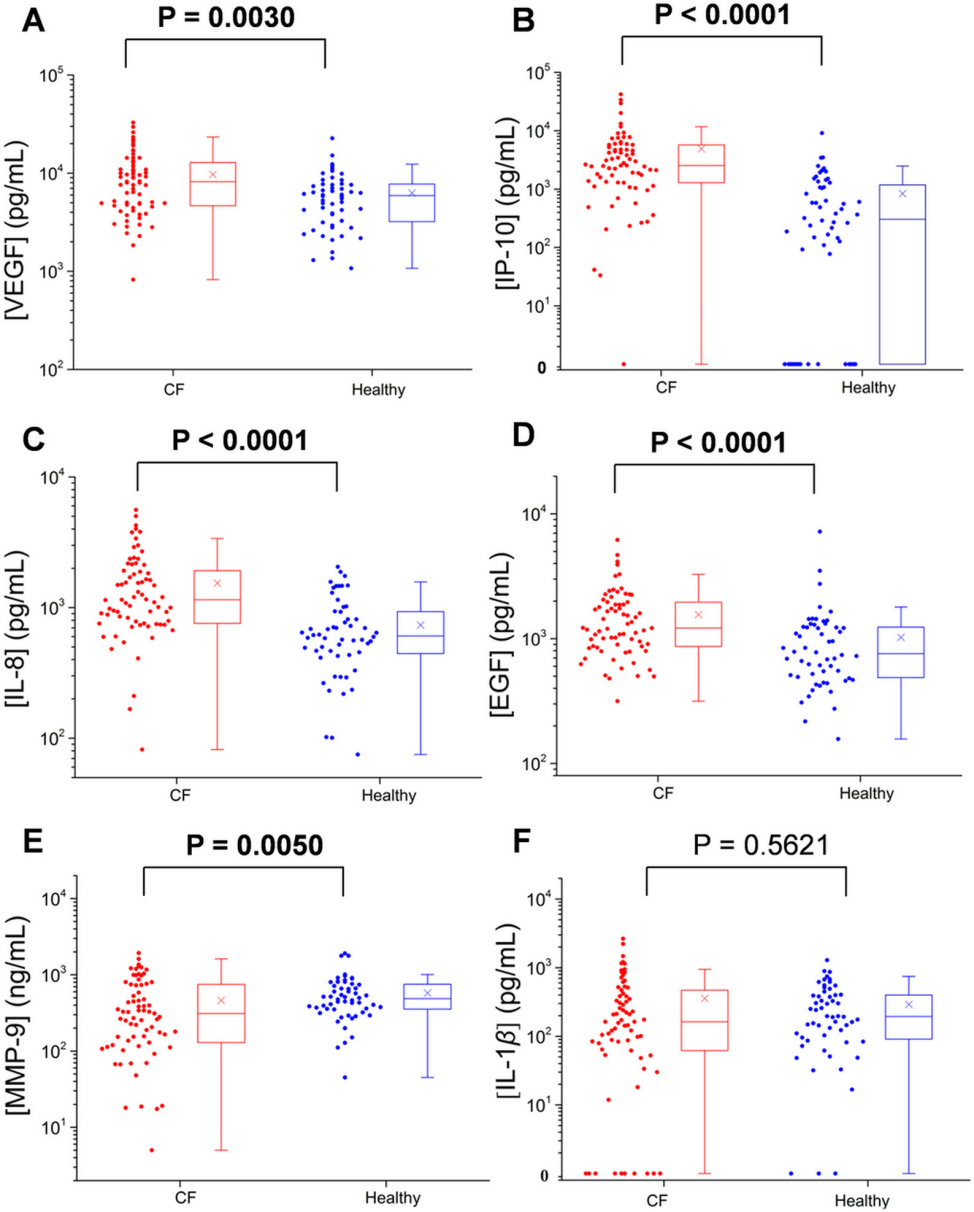
The levels of different proteins in the saliva supernatants of patients with CF (n = 71) and healthy subjects (n = 56) tested by the fiber microarray. The levels of VEGF (**A**), IP-10 (**B**), IL-8 (**C**), and EGF (**D**) in patients with CF were significantly (*P* < 0.005) elevated compared with those from healthy subjects. The levels of MMP-9 (**E**) were significantly lower in patients with CF compared with healthy subjects (*P* = 0.005). IL-1*β* (**F**) was not significantly different (*P* > 0.1) between the two groups.

**Fig 2:**
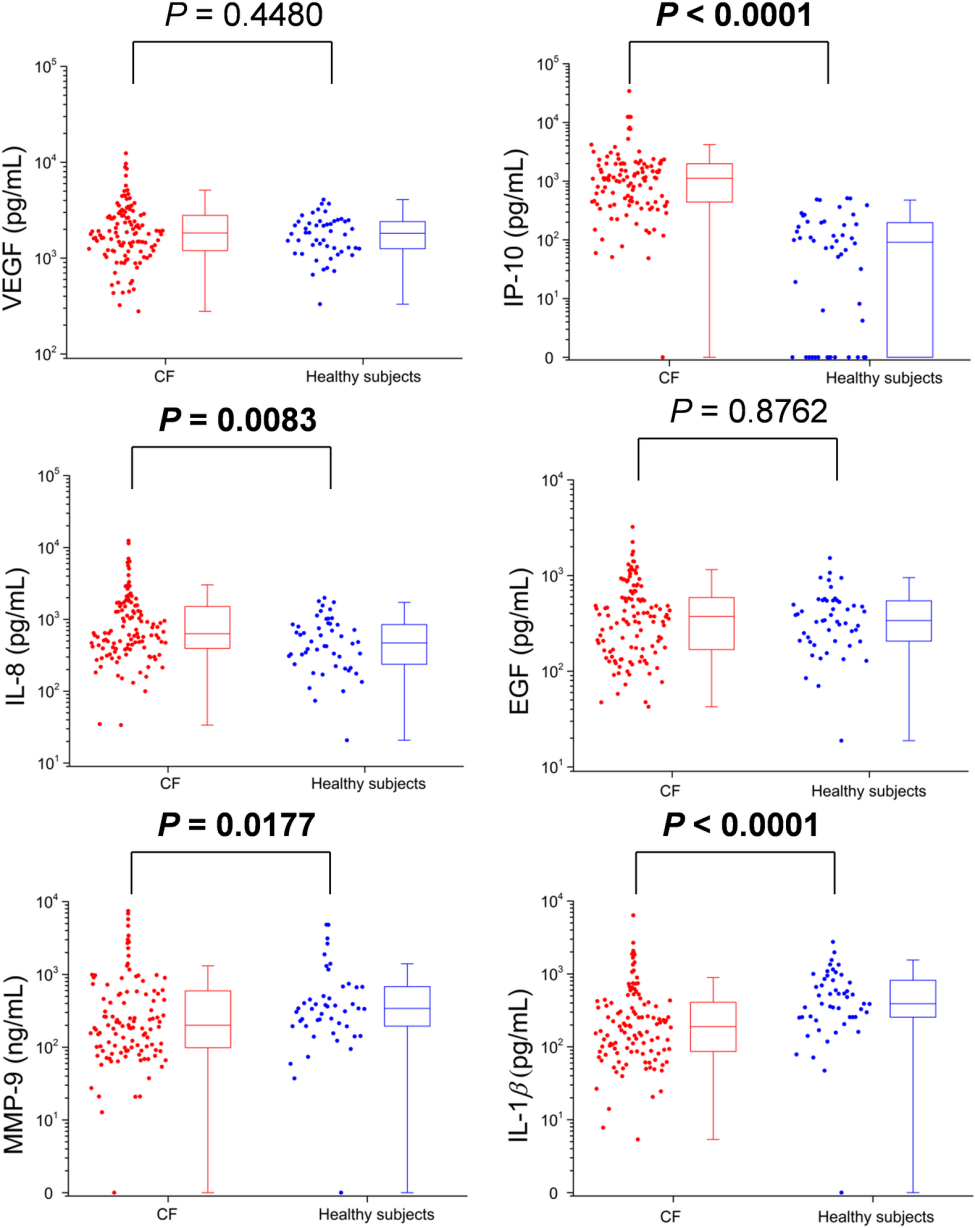
The concentrations of six proteins in the saliva supernatants collected from CF patients (n = 117) and healthy subjects (n = 50) tested by the SDReader. The levels of IP-10 and IL-8 in CF patients were significantly (*P* < 0.01) elevated compared with those from healthy subjects. On the contrary, the levels of MMP-9 and IL-1*β* were significantly lower in CF patients (*P* < 0.02). VEGF and EGF were not significantly different (*P* > 0.1) between the two groups.

The salivary protein biomarker results of patients with CF from both cohorts were first compared with those of healthy subjects. The statistical results from each group are listed in Tables 3 and 4, and the distributions of participants (and boxplots) are shown in Figs 1 and 2.

**Table 3:** Statistical results for six proteins measured in patients with CF and healthy control subjects tested by the fiber microarray. Protein levels in the different groups are presented as median (25th–75th percentile). The statistical results of the same groups have been reported in another format in a previous publication [29]).

Proteins (pg/mL)	Patients with CF (n = 71)	Healthy subjects (n = 56)	Median ratio (CF/HS)	*P* Value
VEGF	8200 (4663–12 850)	5925 (3180–7760)	1.38	**0.0030**
IP-10	2547 (1289–5729)	305 (0–1236)	8.35	**< 0.0001**
IL-8	1150 (758–1918)	607 (439–935)	1.89	**< 0.0001**
EGF	1216 (867–1962)	757 (484–1241)	1.61	**< 0.0001**
MMP-9^*^	312 (129–748)	487 (353–756)	0.64	**0.0050**
IL-1*β*	163 (62–470)	195 (89–402)	0.84	0.5621

^*^: Concentrations in ng/mL.

**Table 4:** Statistical results for six proteins measured in patients with CF and healthy control subjects by the SDReader. Protein levels in the different groups are presented as median (25th–75th percentile).

Proteins (pg/mL)	Patients with CF (n = 117)	Healthy subjects (n = 50)	Median ratio (CF/HS)	*P* Value
VEGF	1831 (1197–2844)	1817 (1231–2417)	1.00	0.4480
IP-10	1118 (434–1989)	91 (0–198)	12.29	**< 0.0001**
IL-8	633 (391–1515)	471 (234–855)	1.34	**0.0083**
EGF	374 (167–620)	339 (206–547)	1.10	0.8762
MMP-9^*^	201 (95–601)	341 (195–698)	0.59	**0.0177**
IL-1β	189 (86–416)	390 (255–827)	0.48	**< 0.0001**

^*^: Concentrations in ng/mL.

For the samples tested by the fiber microarray, the levels of VEGF, IP-10, IL-8, and EGF were significantly elevated in patients with CF as compared with healthy subjects (*P* < 0.005). The median levels of VEGF, IP-10, IL-8, and EGF in patients with CF were increased by factors of 1.38, 8.35, 1.89, and 1.61, respectively. On the contrary, the levels of MMP-9 were significantly lower in patients with CF compared with healthy subjects (*P* = 0.005). There was no statistically significant difference in levels of IL-1*β* between groups.

For the samples tested by the SDReader, patients with CF in this study had significantly elevated levels of IP-10 and IL-8 (*P* < 0.01). To be noted, the median level of IP-10 in patients with CF was increased by a factor of 12.29. On the contrary, the levels of MMP-9 and IL-1*β* were significantly lower in patients with CF compared with healthy subjects (*P* < 0.02). There was no statistically significant difference in levels of VEGF and EGF between groups.

### Protein Levels in Adults with CF and Children with CF

**Fig 3:**
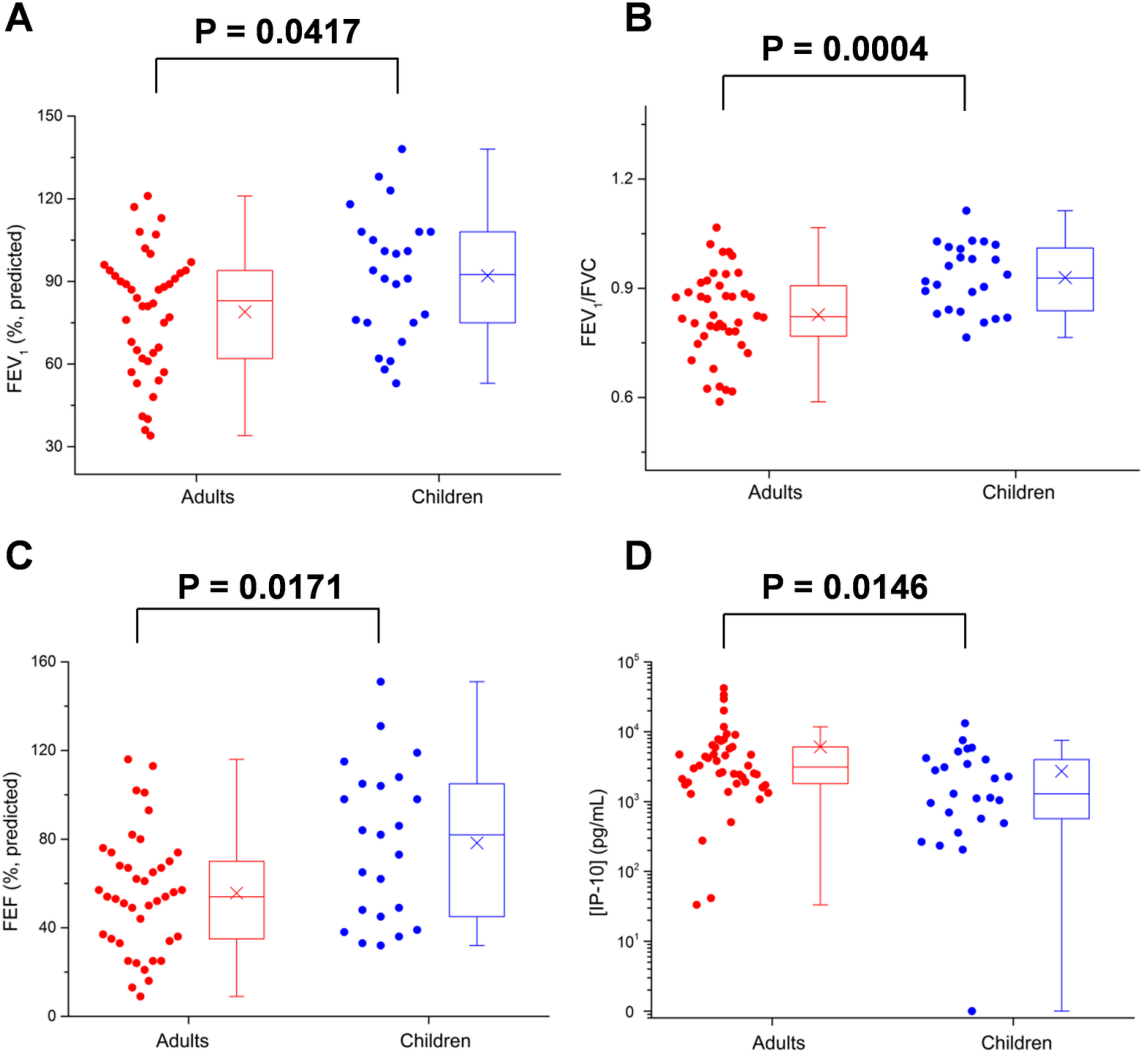
Distributions and boxplots of FEV_1_, FEV_1_/FVC, FEF, and IP-10 levels in adults (n = 46) and children (n = 25) with CF tested by the fiber microarray. (**A**) FEV_1_ values (%, predicted) of adults and children with CF. (**B**) FEV_1_/FVC values of adults and children with CF. (**C**) FEF values (%, predicted) of adults and children with CF. (**D**) Levels of IP-10 in adults and children with CF. Compared with children, adults with CF recruited in this study had significantly lower (*P* < 0.05) FEV_1_, FEV_1/_FVC, and FEF, which demonstrated lower lung function. The levels of IP-10 were significantly elevated (*P* < 0.05) in adults compared to children.

The protein levels in children (age < 18 years) with CF were also compared with those in adults with CF. For the samples tested by the fiber microarray, compared with children, adults with CF in this study (n = 46, 65%) had lower lung function (FEV_1_% predicted: 83 [62–94] vs 93 [75–108], *P* = 0.0417; FEV_1_/FVC 0.82 [0.76–0.91] vs 0.93 [0.84–1.01], *P* = 0.0004; FEF % predicted 54 [35–72] vs 82 [45–105], *P* = 0.0171), a higher incidence of *Pseudomonas aeruginosa* (PA) (80% vs 36%), and a similar incidence of methicillin-resistant *Staphylococcus aureus* (MRSA) (50% vs 52%). The median level of IP-10 in adults with CF was increased by a factor of 2.41 as compared with that in children (*P* = 0.0146). The distributions and boxplots of FEV_1_, FEV_1_/FVC, FEF, and IP-10 levels of patients with CF are shown in Fig 3. The detailed results are listed in Table 5.

**Table 5:** Characteristics of adults and children with CF tested by the fiber microarray.

Characteristics	Adults (age ≥ 18) (n = 46)	Children (age < 18) (n = 25)	*P* Value
Mean age, yr (range)	29 (18–66)	13 (7–17)	
Gender, female (%)	27 (59)	12 (48)	
Genotype			
F508del homozygous (%)	14 (30)	12 (48)	
F508del heterozygous (%)	21 (46)	10 (40)	
Other (%)	11 (24)	3 (12)	
Median FEV_1_% predicted (25%–75%)	83 (62–94)^*^	93 (75–108)^†^	**0.0417**
Median FEV_1_/FVC (25%–75%)	0.82 (0.76–0.91)^*^	0.93 (0.84–1.01)^†^	**0.0004**
Median FEF% predicted (25%–75%)	54 (35–72)^‡^	82 (45–105)^§^	**0.0171**
No. of patients			
Without MRSA and PA (%)	4 (9)	7 (28)	
With MRSA alone (%)	5 (11)	9 (36)	
With PA alone (%)	19 (41)	5 (20)	
With both MRSA and PA (%)	18 (39)	4 (16)	
Protein concentrations			
VEGF (pg/mL)	9043 (4711–12 929)	7612 (4228–13 031)	0.5863
IP-10 (pg/mL)	3127 (1792–6151)	1299 (531–4100)	**0.0146**
IL-8 (pg/mL)	1091 (790–1891)	1256 (742–2182)	0.8624
EGF (pg/mL)	1279 (903–1895)	1216 (791–2106)	0.8343
MMP-9 (ng/mL)	266 (116–621)	328 (166–837)	0.3723
IL-1*β* (pg/mL)	153 (60–481)	208 (66–534)	0.5779

^*^n = 42.

^†^n = 24.

^‡^n = 41.

^§^n = 23.

For the samples tested by the SDReader, based on the spirometry tests, adults recruited in this study (n = 83, 71%) had significantly lower lung function compared with children (FEV_1_% predicted: 65 [49–90] vs 98 [88–106]; FVC % predicted: 85 [67–100] vs 102 [90–109]; FEV_1_/FVC 0.83 [0.72–0.93] vs 0.99 [0.92–1.04]; FEF % predicted 36 [20–73] vs 93 [68–109]; all *P* ≤ 0.0005). However, no significant difference was observed in the protein profiling results between the two groups. The detailed results are listed in Table A in S1 File.

### Correlations between Proteins in Patients with CF

The correlations among the levels of six proteins in patients with CF were also studied. Based on the Spearman test, many proteins were correlated with each other and the correlations were statistically significant. This result is not surprising since these protein biomarkers are a result of an inflammatory cascade and some correlation would be expected. The detailed results are listed in Tables B and C in S1 File.

### Subgroup Analysis of Patients According to Different Medical Characteristics

The protein levels in CF patients with various clinical presentations were also compared. Patients with at least four of the following clinical criteria were defined as experiencing a CF pulmonary exacerbation: increased cough, increased sputum volume or change in color, fever (> 38.0°C), anorexia or weight loss, fatigue or lethargy, new or increased hemoptysis, new findings on chest examination, increased dyspnea, sinus pain or tenderness, change in sinus discharge, decreased pulmonary function or oxyhemoglobin saturation, or new findings on chest radiograph [31].

For the samples tested by the fiber microarray, patients experiencing a CF pulmonary exacerbation (n = 12) showed elevated levels of all six proteins compared to patients not experiencing exacerbation. Patients with better lung function (FEV_1_% predicted > 80, [n = 40]) had significantly elevated levels of IL-1*β* (*P* = 0.0151). Based on the genotyping results, patients with at least one F508 deletion (F508del) (n = 57) had significantly elevated levels of MMP-9 (*P* = 0.0199).

Based on the information about bacterial infections, patients with methicillin-resistant *Staphylococcus aureus* (MRSA, n = 36) showed elevated levels of all six proteins as compared to patients without. The elevations of MMP-9 and IL-1*β* were significant (*P* = 0.0304 and 0.0043, respectively). Patients with *Pseudomonas aeruginosa* (PA) had significantly lower levels of MMP-9 and IL-1*β* (*P* = 0.0041 and 0.0042, respectively) as compared to patients without. The detailed results are listed in Table D in S1 File.

Specifically regarding MRSA and PA infections, 11 patients recruited in this study had neither MRSA nor PA infection, 14 had MRSA alone, 22 had PA alone, and 22 had both. Patients with PA alone had significantly lower levels of MMP-9 and IL-1*β* as compared to all other three groups (*P* = 0.0085 (MMP-9) and 0.0020 (IL-1*β*) for patients with neither MRSA nor PA, *P* = 0.0015 (MMP-9) and 0.0010 (IL-1*β*) for patients with MRSA alone, *P* = 0.0116 (MMP-9) and 0.0007 (IL-1*β*) for patients with both MRSA and PA). The detailed results are shown in Table E in S1 File.

For the samples tested by the SDReader, patients experiencing a CF pulmonary exacerbation (n = 37) showed significantly elevated levels of IP-10 compared to patients not experiencing exacerbation (*P* = 0.0295). Patients with sinusitis had elevated levels of all proteins compared with patients without. Furthermore, the elevations of IL-8 and IL-1*β* were statistically significant. The detailed results are listed in Table F in S1 File.

### Correlations with Clinical Assessments

The possibility of using these protein biomarkers for POC diagnostics was also explored by assessing the correlations between protein profiling results with both spirometry measurements and evaluations made by clinicians. For the samples tested by the fiber microarray, the levels of IP-10 (negatively) and IL-1*β* (positively) significantly correlated with FEV_1_ (r = −0.3029, *P* = 0.0158 and r = 0.2997, *P* = 0.0170, respectively). In addition, the level of IP-10 negatively correlated with FEV_1_/FVC (r = −0.2496, *P* = 0.0485). The severity of individual patients was semi-quantitatively evaluated by the clinicians with a value ranging from 1 to 4 (1: mild, 2: moderate, 3: mod-severe, 4: severe). The level of IL-1*β* negatively correlated with disease severity (r = −0.3505, *P* = 0.0049) and achieved statistical significance. The detailed results are listed in Table 6.

**Table 6:** Correlation of protein levels with clinical assessments of disease severity tested by the fiber microarray (n = 63).

Correlation	VEGF	IP-10	IL-8	EGF	MMP-9	IL-1*β*
FEV_1_	−0.1761 (0.1674)	**−0.3029 (0.0158)**	−0.1051 (0.4126)	−0.1107 (0.3879)	+0.2322 (0.0671)	**+0.2997 (0.0170)**
FEV_1_/FVC	−0.2200 (0.0832)	**−0.2496 (0.0485)**	−0.0721 (0.5743)	−0.1270 (0.3211)	+0.1697 (0.1835)	+0.1904 (0.1350)
FEF	−0.1919 (0.1319)	−0.2212 (0.0815)	−0.0922 (0.4724)	−0.1440 (0.2601)	+0.1594 (0.2120)	+0.2314 (0.0681)
CF Severity^*^	−0.0737 (0.5662)	0.0691 (0.5905)	−0.0187 (0.8847)	−0.0593 (0.6442)	−0.2310 (0.0686)	**−0.3505 (0.0049)**

^*^CF severity: a semi-quantitative evaluation of individual patients by the clinicians, ranging from 1 to 4 (1: mild, 2: moderate, 3: mod-severe, 4: severe).

For the samples tested by the SDReader, based on the 108 CF patients with available spirometry data, the level of IP-10 significantly correlated with FEV_1_ (r = −0.3029, *P* = 0.0158), FEV_1_/FVC (r = −0.2206, *P* = 0.0218), FEF (r = −0.2362, *P* = 0.0139), and disease severity (r = +0.2056, *P* = 0.0262). The detailed results are listed in Table 7.

**Table 7:** Correlation of protein levels with clinical assessments of disease severity tested by the SDReader (n = 108).

Correlation	VEGF	IP-10	IL-8	EGF	MMP-9	IL-1β
FEV_1_	−0.1099 (0.2575)	**−0.1978 (0.0401)**	−0.1003 (0.3019)	−0.0857 (0.3781)	−0.0600 (0.5402)	−0.1117 (0.2292)
FEV_1_/FVC	−0.0767 (0.4301)	**−0.2206 (0.0218)**	−0.0591 (0.5434)	−0.0577 (0.5528)	−0.0141 (0.8850)	−0.0227 (0.8160)
FEF	−0.1033 (0.2873)	**−0.2362 (0.0139)**	−0.0965 (0.3207)	−0.0776 (0.4248)	−0.0447 (0.6462)	−0.0661 (0.4970)
CF Severity^*^	−0.0616 (0.5097)	**0.2056 (0.0262)**	0.1489 (0.1091)	0.0716 (0.4433)	0.0149 (0.8736)	0.0948 (0.3093)

^*^CF severity: a semi-quantitative evaluation of individual patients by the clinicians, ranging from 1 to 4 (1: mild, 2: moderate, 3: mod-severe, 4: severe).

## Discussion

Whole saliva, produced by the major and minor salivary glands, contains many potential diagnostic targets including proteins, small molecules, bacteria, and viruses [32]. Previous research demonstrated that proper sample handling during and after whole saliva collection is essential for downstream protein analysis [33, 34]. The optimal protocol for protein quantification was found to be: collecting whole saliva on ice, centrifuging at 4°C to remove bacteria and mammalian-derived cells, and storing the supernatant at −80°C until analysis [34].

Due to the previously reported elevations of these markers in sputum samples collected from patients with inflammatory disease, we hypothesized that similar elevations would be observed in the saliva samples collected from patients with CF as compared with healthy subjects [20, 21, 35]. However, our study did not focus on sputum analysis as a comparison. In a separate paper involving a cohort of patients with asthma, our colleagues demonstrated no significant correlation between levels of salivary analytes versus levels from nasal lavage fluid [36]. This suggests the oral and upper airway compartments are distinct and we propose the comparison likely exists between the oral and lower airway compartments of CF patients. The agreement in trends from both cohorts in this study reported here reflects distinct findings from sputum reported elsewhere [20, 21, 32, 37]. For the samples tested by the fiber microarray, the significant elevations of VEGF, IP-10, IL-8, and EGF in patients with CF agreed with our expectations from CF sputum studies. Note, the median level of IP-10 in patients with CF was increased by a factor > 8 compared to that in healthy subjects. Contrary to sputum, the levels of MMP-9 and IL-1*β* were lower in the patients with CF as compared to healthy controls. The reasons for these findings need to be further elucidated but considering the dramatic and statistically significant (*P* ≤ 0.005) (other than IL-1*β* in first fiber microarray cohort) differences among CF patients and between CF and healthy patients, VEGF, IP-10, IL-8, EGF, and MMP-9, IL-1*β* show potential as protein markers for CF research and monitoring. The absolute concentrations from the SDReader were different with those from the fiber microarray. However, the trends between different groups agreed well.

Due to the wide distributions of these biomarkers’ levels in both CF patients and healthy subjects, the protein ranges in these two groups overlapped with one another. The average (or median) values for the levels between patients with CF and healthy subjects were often pronounced; however, the value of any diagnostic technique must be able to provide information for individual patients. For some biomarkers, the difference between patients with CF and healthy subjects was large in this study. For example, of the samples tested by both platforms, the range of IP-10 in patients with CF (1289−5729 or 434–1989 pg/mL, quartiles) was distinct from that in the healthy subjects (0−1236 or 0–198 pg/mL, quartiles), which suggests IP-10 may serve as an effective biomarker for CF monitoring. Ultimately, a panel of protein biomarkers will likely prove to be more informative than a single marker.

For all of the patients with CF recruited in this study, spirometry measurements demonstrated that adults had worse lung function than children (significantly lower FEV_1_, FEV_1_/FVC, and FEF). Of the samples tested by the fiber microarray, adults with CF had higher salivary levels of VEGF, IP-10, and EGF and lower levels of IL-8 and MMP-9 than children; however, only the elevation of IP-10 from the fiber microarray cohort was statistically significant. These trends agree with the results from the comparison of patients with CF and healthy subjects: individuals with worse lung function showed elevated levels of VEGF, IP-10, IL-8, and EGF, as well as lower levels of MMP-9 and IL-1*β* in their saliva. However, no significant difference was observed in the samples tested by the SDReader despite similar trends.

The results of the Spearman test of the correlations between the six protein markers showed that most of the proteins positively correlated with each other. There were close correlations amongst the angiogenesis factor VEGF that has been reported to induce the augmentation of the T cell chemoattractant chemokine IP-10 and markers of innate/myeloid immune activation (e.g. IL-1*β* vs IL-8) [38]. The lack of correlation between IP-10 and IL-1*β*—two biologically unrelated inflammatory markers—demonstrated that these correlations are disease-specific rather than being generalized outcomes of increased inflammatory effects in the oral cavity.

Differences in protein levels were also observed in the subgroup analysis of patients with different clinical presentations. Patients experiencing acute CF exacerbation were reported to have elevated levels of inflammatory markers in their sputum samples [39, 40]. The same trends were also observed in the saliva supernatants. Among patients from the fiber microarray cohort, the median levels of the six proteins in patients at acute exacerbation were increased by factors of 1.66, 2.34, 1.92, 1.49, 1.20, and 1.21, respectively. However, none of these elevations achieved statistical significance, probably due to the wide distributions of the protein levels and/or the limited number of patients at acute exacerbation (n = 12). Similar trends were observed from the SDReader cohort, but this time IP-10 was significantly elevated for those experiencing an acute exacerbation (Table F in S1 File). The addition of IP-10 to a panel of clinical and biologic markers could help CF care providers more specifically identify pulmonary exacerbations and reduce inappropriate variations in disease management.

Spirometry measurements (such as FEV_1_, FEV_1_/FVC, and FEF) are the most common pulmonary function tests used to assess the condition of patients with respiratory diseases. FEV_1_ was reported to be correlated with various protein markers in sputum [40, 41]. For patients with worse lung function in this study (characterized by lower FEV_1_ values), statistical significance was only achieved with lower levels of IL-1*β* observed in the fiber microarray cohort (shown in Table 5). These results suggest the possibility of using these protein markers in lung function assessment. As a complex biological fluid, whole saliva reflects both local and systemic inflammation, and may explain the difference in observation from CF sputum and systemic circulation [42].

F508del is the most common CF mutation in North American populations, accounting for about 70% of CF mutations [43]. Based on genotyping, 81% of the patients with CF in this study were identified to carry at least one F508del allele. The patients with F508del showed elevated levels of VEGF, IL-8, EGF, MMP-9 and IL-1*β* as compared to other CF patients. These findings are not surprising given the association F508del has with more advanced lung disease. Other CF specific biomarkers may further expand these findings. Due to the time limitation, this information was not collected from the samples tested by the SDReader.

There is a large, increasingly well characterized, heterogeneous microbial community in the CF airway with known antibiotic resistance patterns that will provide ample opportunity to verify diagnostic capability. Furthermore, earlier knowledge of bacterial infection and identification of antibiotic resistance would enhance success in eradication of difficult-to-treat infections in CF [3]. Information about bacterial infections including MRSA, PA, and Burkholderia cepacia complex (BCC) were collected in this study. In our fiber microarray cohort, significantly elevated levels of MMP-9 and IL-1*β* were observed in patients with MRSA. By contrast, the levels of MMP-9 and IL-1*β* were significantly lower in patients with PA only. This observation is contrary to findings from sputum [37, 44] and a potentially very interesting one and also reflects the distinctiveness of the oral cavity and lower airway. Systemic IL-1*β* levels may not correlate with lower airway levels. All six protein levels were predictably elevated during exacerbations, triggered by a viral infection greater than 50% of the time [45]. The observation of lower levels of IL-1*β* in saliva from patients chronically infected with PA, and not infected with MRSA, may reflect a deficiency in innate defense or attenuation of systemic IL-1*β* among patients chronically infected with PA versus those with acute infection with other organisms [46]. A recent study reported on NLRC4 (Nods-Like Receptor family, CARD domain containing 4) associated inflammasome activation by the type 3 secretion system (T3SS) [47], which typically increases IL-1*β* and lung neutrophil recruitment through IL-18. The authors demonstrated a pathway that leads to IL-18 mediated down regulation of IL-17 secretion and impaired bacterial clearance. Future analysis should include measure of IL-18 to observe cytokine response from shared inflammatory pathway promoted by inflammasome [48]. Defective autophagy has also been implicated in pathogenesis of CF lung disease and may be playing role in dyregulated inflammatory response [49]. Due to the limited number of patients with BCC (4 patients total), the corresponding comparisons were not performed. There is a delay between newly acquired lower airway bacterial infections and positive sputum cultures. Salivary biomarkers from CF patients at risk for new infections, like PA and MRSA, may help identify and eradicate these acutely acquired disease-altering infections.

The SDReader adds additional value by providing POC biomarker data in real time and the device is capable of targeting other potentially more predictive biomarkers and remains a goal for the future (30). Due to its property to characterize the sample donor’s health condition at the time of collection, saliva has been termed a “real-time” indicator [18]. We propose that rapid salivary diagnostics would improve CF patient care. To test this assumption, we have successfully developed an integrated, portable platform that enables automated profiling of up to 10 salivary proteins in just 70 min [30]. Based on a fully automated assay process and the noninvasive collection of saliva samples, this platform can be used by personnel with minimal training and shows great potential for low-resource settings. In this study, the platform was deployed in the CF clinic at Boston Children’s Hospital. Collection and analysis of fresh saliva samples from patients with CF were successfully carried out on-site. The rapid assay has provided timely protein profiling information, which may assist with CF research and monitoring. By incorporating the results described here with the POC device described in other work, individuals may be able to self-monitor these protein levels using an easy to obtain sample specimen.

In order to explore the potential use of saliva in POC diagnostics and patient self-monitoring in settings with limited medical resources, the correlations between protein levels and four disease evaluation methods were also studied. The level of IP-10 significantly negatively correlated with FEV_1_ and FEV_1_/FVC. The level of IL-1*β* significantly correlated with FEV_1_ (positively) and CF severity (negatively) as diagnosed by clinicians. While the correlations discussed here are based on the group of patients with different clinical characteristics, the ultimate value of such biomarker measurements will be for individual patients. The preliminary results presented here suggest that salivary protein biomarkers have the potential to be used in place of or strengthen conventional clinical evaluation methods. Such measurements may be able to identify individuals at greatest risk for adverse outcomes or significant acute exacerbations. Finally, it is important to note that individuals may vary in their baseline levels of these biomarkers, as is evident from the rather large ranges obtained in healthy individuals. Baseline variations may be due to differences in underlying genetics, home environment, or other factors. Consequently, measuring the levels in individuals over time may be a better indicator of a change in disease state or management. Due to the limited number of patients and clinician subjectivity, our current results do not provide sufficient predictive ability for clinical decisions to be made regarding individual patients, as is generally the case with novel biomarkers in the early stages of investigation. However, this work could be a cornerstone for future studies exploring the longitudinal behavior of salivary protein markers using more sophisticated analyses and long-term follow-up. Combining current clinical data with biomarker analyses may lead to better therapeutic decision making by clinicians. The major limitation of our study is the absence of sputum analysis for comparison and something we will address in future work. The two separate platforms allowed us to demonstrate similar trends in biomarker levels but the absolute levels due to these unique assays complicates analysis. A study using one platform to measure biomarker levels in a greater number of CF patients with sputum for comparison will be needed to bolster our observations here.

## Supporting Information

S1 File Table A. Characteristics of adults and children with CF tested by the SDReader. Table B. Correlations between different protein markers in patients with CF tested by the fiber microarray. Table C. Correlations between different protein markers in patients with CF tested by the SDReader. Table D. Median protein levels in different subgroups of patients with CF tested by the fiber microarray. Table E. Median protein levels in different subgroups of patients with MRSA and PA infections tested by the fiber microarray. Table F. Median protein levels in different subgroups of patients with CF tested by the SDReader. (DOCX)
