# Supplementary material for: Correlations of Salivary Biomarkers with Clinical Assessments in Patients with Cystic Fibrosis
Source: PLoS One. 2015 Aug 10;10(8):e0135237. doi: 10.1371/journal.pone.0135237 (PMC4530931; doi:10.1371/journal.pone.0135237)
Supplement: S1 File — (DOCX) [file pone.0135237.s001.docx]

**Correlations of Salivary Biomarkers with Clinical Assessments in Patients with Cystic Fibrosis**

Shuai Nie^1^, Huaibin Zhang^1^, Kathryn M. Mayer^1^, Frank G. Oppenheim^2^, Frédéric F. Little^3^, Jonathan Greenberg^4^, Ahmet Z. Uluer^4, *^, and David R. Walt^1, *^

^1^Department of Chemistry, Tufts University, Medford, Massachusetts, United States of America;

^2^Goldman School of Dental Medicine, Boston University, Boston, Massachusetts, United States of America;

^3^School of Medicine, Boston University, Boston, Massachusetts, United States of America;

^4^Division of Respiratory Diseases, Boston Children’s Hospital and Brigham & Women’s Hospital, Harvard Medical School, Boston, Massachusetts, United States of America;

* Corresponding author

E-mail: [ahmet.uluer@childrens.harvard.edu](mailto:ahmet.uluer@childrens.harvard.edu) (AZU)

E-mail: [david.walt@tufts.edu](mailto:david.walt@tufts.edu) (DRW)

Table of Contents

| 1. Questionnaire of CF patients……….............................................................................. | 3 |
| --- | --- |
| 1. Characteristics of adults and children with CF tested by the SDReader (S1 Table)...... | 10 |
| 1. Correlations between different proteins tested by the fiber microarray (S2 Table)...… | 11 |
| 1. Correlations between different proteins tested by the SDReader (S3 Table)...………. | 11 |
| 1. Protein levels in subgroups tested by the fiber microarray (S4 Table)……………….. | 12 |
| 1. Protein levels in MRSA and PA tested by the fiber microarray (S5 Table)………….. | 13 |
| 1. Protein levels in subgroups tested by the SDReader (S6 Table)……………………… | 14 |
|  |  |
|  |  |
|  |  |

**Section** **I:** **This** **data** **should** **be** **obtained** **prior** **to** **saliva** **collection** **and** **must** **be** **entered** **into** **the** **database** **immediately** **in** **order** **to** **create** **a** **label** **for** **the** **saliva** **collection** **container.**

(Today’s date *(MM/DD/YYYY)* captured automatically by website)

Patient’s Initials: _______________________

DOB: / / *(MM/DD/YYYY)* (Age calculated by website)

Gender: 1 Male 2 Female 3 Transgender

Ethnicity: Is the patient of Hispanic (Latino/Mexican/Puerto Rican/Central or South American) origin or descent?

1 No, not Hispanic 2 Yes, Hispanic 9 Don't Know / No Response

Race *(check* *all* *that* *apply)*:

1 American Indian/Alaskan Native 4 Native Hawaiian/Pacific Islander

2 Asian 5

3 Black 8

White

Other (SPECIFY) ______________

9 Don't Know / No Response

Study Arm: 1 Case 0 Control

(Subject number and sample numbers/barcodes created by website)

Subject # created by website/barcode system: Coordinator/Interviewer’s initials:

**Section** **II:** **Sample** **collection** **Saliva** **collection:**

Collection start time: . Collection end time: .

*(24:00* *hr:min)*

*(24:00* *hr:min)*

(Total volume measured and entered by lab personnel) (Flow Rate(ml/min) calculated by website)

Number of hours since last ate/drank anything: .

**Section** **III:** **Questions** **should** **be** **asked** **after** **saliva** **collection,** **but** **can** **be** **entered** **into** **database** **afterwards**

1. Height (inches)

2. Weight (pounds)

**Clinical** **Data** .

.

3a. If >=18 years old, BMI (kg/m2): .

3b. If <18 years old, BMI (%): . %

4. FEV1 5. FVC 6. FEF

7. Sweat chloride 8. CF Genotype

. L . L . L

.

1 DeltaF508 1 DeltaF508

Not Done Not Done Not Done

mEq/L Not Done

2 Unidentified 3 Other 0 N/A 2 Unidentified 3 Other 0 N/A

**Saliva** **collection** **questions**

9. Are you currently treated by any type of health

professional for any oral conditions including 1 Yes 0 No 9 Unknown caries or periodontal disease?

9a. If yes, specify what type: __________________________________

10. When did you last see a dentist or dental hygienist for tooth cleaning (in weeks or

months)? Weeks Months

11. At what interval do you see a dental professional for oral check-ups (in months)?

Months 12.How many times do you brush your teeth per day?

13.How many times do you floss per week? 14. Do you mouthrinse?

1 Daily 2 Often 3 Occasionally 4 Rarely 5 Never Brand: __________________________________

15.Do you feel you have a healthy mouth? 1 Yes 0 No 9 Unknown 15a. If not, what troubles do you have with your teeth, gums, or oral soft tissues?

16. Do you currently suffer from any oral infections (bacterial or viral) in your mouth? 1 17. Have you accidentally bitten your cheek

mucosa recently? 1

18. Do you have any pain chewing? 1

19. Do you feel that you have painful and/or

swollen and/or bleeding gums? 1 20. Do you bleed or see small amounts of blood

upon rinsing your mouth after brushing? 1 21. Do you feel that you should see an oral health professional for something bothering you in your 1 mouth?

**Oral** **Exam**

22.Number of teeth in oral cavity:

Yes 0 No 9

Yes 0 No 9

Yes 0 No 9

Yes 0 No 9

Yes 0 No 9

Yes 0 No 9

Unknown

Unknown

Unknown

Unknown

Unknown

Unknown

23.Full or partial dentures: 0 Own teeth 1 None 2 Full 3 Partial 24. Teeth clean or covered with plaque deposits? 0 Clean 1 Plaque Deposits 25. Gums pale or reddish? 0 Pale 1 Reddish

26. Cheek mucosa normal or abnormal? 0 Normal 1 Abnormal 27. Tongue mucosa normal or abnormal? 0 Normal 1 Abnormal

28. Needs to be seen by a dental professional? 1 Yes 0 No 9 Unknown **Oral** **Disease** **History**

29. Oral Disease?

29a. If yes, Periodontal Disease/Gingivitis? 29b. If yes, date last diagnosed with Periodontal

Disease/Gingivitis

29c. If yes, Oral Cancer?

29d. If yes, date last diagnosed with oral cancer 29e. If yes, dental caries/cavities?

29f. If yes, date last diagnosed with dental caries/cavities

30. Tonsillectomy?

30a. If yes, date of tonsillectomy 31. Reflux Disease?

31a. If yes, date of diagnosis

1 Yes 0 No 1 Yes 0 No

1 Yes 0 No

1 Yes 0 No

1 Yes 0 No

1 Yes 0 No

9 Unknown 9 Unknown

9 Unknown

9 Unknown

9 Unknown

9 Unknown

**Social** **History**

32. Do you drink alcohol? 1 Yes 0 No 9 Unknown

32a.If yes, number of drinks per week?

1 0-3 2 4-7 3 >7

33. Smoking history? 1 Yes 0 No 9 Unknown

33a. If yes, what? 1 Cigarettes 33b. If yes, current smoker?

2 Cigars 3 Chewing tobacco

1 Yes 0 No 9 Unknown

33c. If yes, number of packs per day?

33d. If yes, how many years of smoking?

33e. If yes, when was last cigarette?

If current smoker, give answer in hours: .

If former smoker, give answer as month/year: /

34. Recreational inhalants? 1 Yes 0 No 9 Unknown 34a. If yes, what?

1 Marijuana 2 Heroin 3 Cocaine/crack 8 Other (specify): __________ 34b. If yes, when was your last exposure (in days, weeks, or years)?

Days Weeks Years

35. Family history of asthma?

36. Family history of allergic rhinitis/hayfever?

1 Yes 0 No 1 Yes 0 No

9 Unknown 9 Unknown

**Other** **Medical** **History**

37. Pancreatic status? 1 Sufficient 2 Insufficient 0 N/A

38. CF related diabetes?

39. CF related liver disease? 40. G-tube?

41. CF related arthritis? 42. Hypertension? OB/GYN History

43. Do you think you might be pregnant?

Dermatologic History

44. Eczema? 45. Dermatitis?

Other

1 Yes 0 No 1 Yes 0 No 1 Yes 0 No 1 Yes 0 No 1 Yes 0 No

8 N/A 1 Yes 0 No

1 Yes 0 No 1 Yes 0 No

9 Unknown 9 Unknown 9 Unknown 9 Unknown 9 Unknown

9 Unknown

9 Unknown 9 Unknown

46. Have you been hospitalized in the past 3 months?

1 Yes 0 No 9 Unknown

**Pulmonary** **History**

47. Asthma / Airway Hyperactivity? 1 Yes 0 No 9 Unknown

48. Allergic rhinitis/”hay fever”? 1 Yes 0 No 9 Unknown Use 0-3 scale for personal ratings: 0=No symptoms; 3=Interferes with daily life

48a. If yes, personal rating: Runny Nose (0-3)

48b. If yes, personal rating: Itchy Nose (0-3)

48c. If yes, personal rating: Sneezing (0-3)

48d. If yes, personal rating: Nasal Congestion (0-3)

48e. If yes, personal rating: Itching/Burning Eyes (0-3)

48f. If yes, personal rating: Tearing/Watery Eyes (0-3)

48g. If yes, personal rating: Redness of the Eyes (0-3)

48h. If yes, personal rating: Itching of the Ears or Palate (0-3)

49. Allergen Immunotherapy? 1 Yes 0 No 9 Unknown 49a. If yes, number of allergens (1 to 8, >8)

1 1 2 2 3 3 4 4 5 5

6 6 7 7 8 8 9 >8

49b. If yes, when started allergy immunotherapy? *(MM/* *DD/YYYY)* / /

50. Nasal Polyposis? 51. Sinusitis?

52. Hemoptysis requiring embolization? 53. Chronic O2 required?

54. ABPA? Chronic Infection:

55. Pseudomonas? 56. MRSA?

57. B. cepacia complex?

58. Nontuberculous mycobacteria?

1 Yes 0 No 1 Yes 0 No 1 Yes 0 No 1 Yes 0 No 1 Yes 0 No

1 Yes 0 No 1 Yes 0 No 1 Yes 0 No 1 Yes 0 No

9 Unknown 9 Unknown 9 Unknown 9 Unknown 9 Unknown

9 Unknown 9 Unknown 9 Unknown

9 Unknown

**Pulmonary** **Exacerbation**

Major Criteria:

59. Decrease in FEV1? 1 60. Decreased oxygen saturation? 1 61. New lobar infiltrate or atelectasis? 1 62. Hemoptysis? 1 Minor Criteria:

63. Increased respiratory rate? 1

64. New or increased adventitial sounds on lung exam? 1

65. Weight loss? 1 66. Increased cough? 1 67. Decreased exercise tolerance or activity level? 1

Yes 0 No 9 Yes 0 No 9 Yes 0 No 9 Yes 0 No 9

Yes 0 No 9

Yes 0 No 9

Yes 0 No 9 Yes 0 No 9 Yes 0 No 9

Unknown Unknown Unknown Unknown

Unknown

Unknown

Unknown Unknown

Unknown

68. Increased chest congestion or change in sputum?

Duration Criteria:

69. Duration of signs/symptoms > 5 days? 70. Do the signs/symptoms above meet the

definition of pulmonary exacerbation (1 major or 2 minor + > 5 days)?

71. Were intravenous antibiotics required?

1 Yes 0 No

1 Yes 0 No

1 Yes 0 No

1 Yes 0 No

9 Unknown

9 Unknown

9 Unknown

9 Unknown

**Current** **Asthma** **Medications**

72. Prednisone? 1 Yes 0 No 9 Unknown 72a. If yes, how much (0-120mg)?

72b. If yes, how often (times per day)?

1 Once per day 2 Twice per day 72c. If yes, for how long in days? 1 0-180

**Other** **Medications**

3 More than twice per day

2 >180

**Please** **list** **all** **medications** **you** **are** **currently** **taking,** **including** **hypertonic** **saline** **solution,** **over** **the** **counter** **medications** **and** **vitamins.**

**If** **you** **need** **additional** **space,** **please** **use** **the** **other** **side** **of** **this** **piece** **of** **paper.**

**END** **OF** **QUESTIONNAIRE**

Date form **first** **entered** on website (MM/DD/YYYY):

Data entry person’s initials:

Date form **double** **entered** on website (MM/DD/YYYY):

Data entry person’s initials:

**S1 Table. Characteristics of adults and children with CF tested by the SDReader.**

| **Characteristics** | **Adults (age ≥ 18)**  **(n=83)** | **Children (age < 18)**  **(n= 34)** | ***P* Value** |
| --- | --- | --- | --- |
| **Mean age, yr (range)** | **32 (18–67)** | **13 (8–17)** |  |
| **Sex, female (%)** | **46 (55)** | **12 (35)** |  |
| **Median FEV_1_% predicted (25%–75%)** | **65 (49–90)*** | **98 (88–106)†** | ****<0.0001**** |
| **Median FVC% predicted (25%–75%)** | **85 (67–100)*** | **102 (90–109)†** | ****0.0005**** |
| **Median FEV_1_/FVC (25%–75%)** | **0.83 (0.72–0.93)*** | **0.99 (0.92–1.04)†** | ****<0.0001**** |
| **Median FEF% predicted (25%–75%)** | **36 (20–73)*** | **93 (68–109)†** | ****<0.0001**** |
| **No. of patients** |  |  |  |
| **Without MRSA and PA (%)** | **4 (9)** | **7 (28)** |  |
| **With MRSA alone (%)** | **5 (11)** | **9 (36)** |  |
| **With PA alone (%)** | **19 (41)** | **5 (20)** |  |
| **With both MRSA and PA (%)** | **18 (39)** | **4 (16)** |  |
| **Protein concentrations** |  |  |  |
| **VEGF (pg/mL)** | **1679 (1170–2668)** | **1899 (1204–2917)** | **0.7919** |
| **IP-10 (pg/mL)** | **1042 (406–1967)** | **1109 (413–1685)** | **0.7916** |
| **IL-8 (pg/mL)** | **639 (325–1341)** | **639 (325–1341)** | **0.7971** |
| **EGF (pg/mL)** | **329 (157–687)** | **348 (146–460)** | **0.4982** |
| **MMP-9 (ng/mL)** | **185 ( 89–585)** | **201 (91–391)** | **0.8800** |
| **IL-1β (pg/mL)** | **126 (83–327)** | **126 (82–380)** | **0.3887** |

^*^n = 75.

^†^n = 33.

**S2 Table. Correlations between different protein markers in patients with CF tested by the fiber microarray.**

|  | IP-10 | IL-8 | EGF | MMP-9 | IL-1*β* |
| --- | --- | --- | --- | --- | --- |
| VEGF | r = 0.5638  (*P* < 0.0001) | r = 0.7176  (*P* < 0.0001) | r = 0.6739  (*P* < 0.0001) | r = 0.4279  (*P* = 0.0002) | r = 0.2311  (*P* = 0.0525) |
|  | IP-10 | r = 0.7508  (*P* < 0.0001) | r = 0.5555  (*P* < 0.0001) | r = 0.3724  (*P* = 0.0014) | r = 0.2417  (*P* = 0.0423) |
|  |  | IL-8 | r = 0.6123  (*P* < 0.0001) | r = 0.6372  (*P* < 0.0001) | r = 0.4918  (*P* < 0.0001) |
|  |  |  | EGF | r = 0.4241  (*P* = 0.0002) | r = 0.1877  (*P* = 0.1171) |
|  |  |  |  | MMP-9 | r = 0.5801  (*P* < 0.0001) |

**S3 Table. Correlations between different protein markers in patients with CF tested by the SDReader.**

|  | IP-10 | IL-8 | EGF | MMP-9 | IL-1*β* |
| --- | --- | --- | --- | --- | --- |
| VEGF | r = 0.4500  (*P* < 0.0001) | r = 0.7296  (*P* < 0.0001) | r = 0.7310  (*P* < 0.0001) | r = 0.5846  (*P* < 0.0001) | r = 0.6561  (*P* < 0.0001) |
|  | IP-10 | r = 0.3716  (*P* < 0.0001) | r = 0.1889  (*P* = 0.0414) | r = 0.2017  (*P* = 0.0292) | r = 0.1525  (*P* = 0.1008) |
|  |  | IL-8 | r = 0.5941  (*P* < 0.0001) | r = 0.7618  (*P* < 0.0001) | r = 0.7765  (*P* < 0.0001) |
|  |  |  | EGF | r = 0.4423  (*P* < 0.0001) | r = 0.5610  (*P* < 0.0001) |
|  |  |  |  | MMP-9 | r = 0.7905  (*P* < 0.0001) |

**S4 Table. Median protein levels in different subgroups of patients with CF tested by the fiber microarray.**

| **Protein**  **(pg/mL)** | **Acute exacerbation** | | | **FEV_1_ (% predicted)** | | | **F508del mutation** | | |
| --- | --- | --- | --- | --- | --- | --- | --- | --- | --- |
|  | No  (n = 59) | Yes  (n = 12) | *P* | > 80  (n = 40) | < 80  (n = 27) | *P* | No  (n = 12) | Yes  (n = 57) | *P* |
| VEGF | 7993 | 13241 | 0.1167 | 7563 | 9115 | 0.5212 | 4808 | 8972 | 0.0889 |
| IP-10 | 2492 | 5821 | 0.1975 | 2448 | 3110 | 0.3632 | 3125 | 2539 | 0.6901 |
| IL-8 | 1084 | 2081 | 0.0995 | 1099 | 1180 | 0.6613 | 819 | 1157 | 0.1584 |
| EGF | 1163 | 1737 | 0.0872 | 1209 | 1148 | 0.9444 | 1015 | 1222 | 0.1997 |
| MMP-9^*^ | 312 | 375 | 0.7218 | 386 | 207 | 0.0524 | 152 | 341 | **0.0199** |
| IL-1*β* | 163 | 197 | 0.6766 | 249 | 87 | **0.0151** | 121 | 209 | 0.1629 |

| **Protein**  **(pg/mL)** | **MRSA** | | | **PA** | | |
| --- | --- | --- | --- | --- | --- | --- |
|  | No  (n = 35) | Yes  (n = 36) | *P* | No  (n = 25) | Yes  (n = 46) | *P* |
| VEGF | 8200 | 8554 | 0.6189 | 8200 | 8145 | 0.7605 |
| IP-10 | 4757 | 5866 | 0.8594 | 1808 | 2595 | 0.1582 |
| IL-8 | 1150 | 1310 | 0.2310 | 1504 | 1058 | 0.3659 |
| EGF | 1148 | 1508 | 0.3333 | 1314 | 1191 | 0.8157 |
| MMP-9^*^ | 189 | 368 | **0.0304** | 728 | 224 | **0.0041** |
| IL-1*β* | 98 | 273 | **0.0043** | 330 | 107 | **0.0042** |

^*^: Concentrations of MMP-9 are in ng/mL.

**S5 Table. Median protein levels in different subgroups of patients with MRSA and PA infections tested by the fiber microarray.**

| Protein  (pg/mL) | PA alone | Neither MRSA nor PA | | MRSA alone | | Both MRSA and PA | |
| --- | --- | --- | --- | --- | --- | --- | --- |
|  | n =24 | n =11 | *P*^*^ | n =14 | *P*^*^ | n =22 | *P*^*^ |
| VEGF | 7808 | 10000 | 0.4508 | 6669 | 0.7538 | 9132 | 0.4517 |
| IP-10 | 3187 | 1808 | 0.1979 | 2296 | 0.5598 | 2520 | 0.9393 |
| IL-8 | 1091 | 1256 | 0.5632 | 1591 | 0.1355 | 990 | 0.6086 |
| EGF | 1086 | 1216 | 0.5167 | 1456 | 0.5398 | 1508 | 0.2793 |
| MMP-9^†^ | 155 | 748 | **0.0085** | 603 | **0.0015** | 341 | **0.0116** |
| IL-1*β* | 72 | 330 | **0.0020** | 335 | **0.0010** | 231 | **0.0007** |

^*^: Compared with the group of “patients with PA alone”.

^†^: Concentrations of MMP-9 are in ng/mL.

**S6 Table. Median protein levels in different subgroups of patients with CF tested by the SDReader.**

| **Protein**  **(pg/mL)** | **Acute exacerbation** | | | **Sinusitis** | | |
| --- | --- | --- | --- | --- | --- | --- |
|  | No  (n = 80) | Yes  (n = 37) | *P* | No  (n = 53) | Yes  (n = 62) | *P* |
| VEGF | 1783 | 1224 | 0.9988 | 1628 | 1923 | 0.3365 |
| IP-10 | 1026 | 1801 | **0.0295** | 1127 | 1054 | 0.8394 |
| IL-8 | 621 | 639 | 0.9500 | 549 | 789 | **0.0199** |
| EGF | 363 | 383 | 0.5853 | 322 | 420 | 0.5742 |
| MMP-9^*^ | 209 | 180 | 0.6458 | 151 | 238 | 0.0705 |
| IL-1*β* | 190 | 169 | 0.7405 | 123 | 236 | **0.0288** |

| **Protein**  **(pg/mL)** | **MRSA** | | | **PA** | | |
| --- | --- | --- | --- | --- | --- | --- |
|  | No  (n = 35) | Yes  (n = 36) | *P* | No  (n = 25) | Yes  (n = 46) | *P* |
| VEGF | 8200 | 8554 | 0.6189 | 8200 | 8145 | 0.7605 |
| IP-10 | 4757 | 5866 | 0.8594 | 1808 | 2595 | 0.1582 |
| IL-8 | 1150 | 1310 | 0.2310 | 1504 | 1058 | 0.3659 |
| EGF | 1148 | 1508 | 0.3333 | 1314 | 1191 | 0.8157 |
| MMP-9^*^ | 189 | 368 | **0.0304** | 728 | 224 | **0.0041** |
| IL-1*β* | 98 | 273 | **0.0043** | 330 | 107 | **0.0042** |

^*^: Concentrations of MMP-9 are in ng/mL.
